# Visual and Spatial Working Memory Abilities Predict Early Math Skills: A Longitudinal Study

**DOI:** 10.3389/fpsyg.2019.02460

**Published:** 2019-11-06

**Authors:** Rachele Fanari, Carla Meloni, Davide Massidda

**Affiliations:** Department of Pedagogy, Psychology, Philosophy, Faculty of Humanities, University of Cagliari, Cagliari, Italy

**Keywords:** active working memory, visual working memory, spatial working memory, early math competence, math skills, longitudinal study

## Abstract

This study aimed to explore the influence of the visuospatial active working memory subcomponents on early math skills in young children, followed longitudinally along the first 2 years of primary school. We administered tests investigating visual active working memory (jigsaw puzzle), spatial active working memory (backward Corsi), and math tasks to 43 children at the beginning of first grade (T1), at the end of first grade (T2), and at the end of second grade (T3). Math tasks were selected according to the children’s age and their levels of formal education: the “Battery for the evaluation of numerical intelligence from 4 to 6 years of age” (BIN 4–6) at T1 to test early numerical competence and the “Test for the evaluation of calculating and problem-solving abilities” (AC-MT 6–11) to test math skills at T2 and T3. Three regression models, in which the predictors were identified through a backward selection based on the use of the Bayesian information criterion (BIC) index, were performed to study the relationship between visual and spatial working memory and math ability at the three points in time. The results show that spatial working memory influences early numerical performance at T1, while early numerical performance is the unique predictor of math performance at T2. At the end of the second grade, the regression model reveals a relationship between math performance and both visual and spatial working memory and the attenuation of the importance of domain-specific predictors. The study depicts the different implications of visual and spatial working memory predictors over the children’s development periods and brings additional evidence to the debate on the relationship between visuospatial working memory and math ability in young children.

## Introduction

The development of early math skills involves domain-specific skills, such as quantity understanding, numbers recognition, counting skills (Geary et al., 2000; Krajewski and Schneider, 2009), and general domain cognitive skills (Fuchs et al., 2005; Passolunghi et al., 2007, 2008; De Smedt et al., 2009). One of the general domain abilities most frequently associated with the development of mathematical skills is working memory (WM) (Hitch, 1978; McLean and Hitch, 1999; Gathercole and Pickering, 2000; Rasmussen and Bisanz, 2005; Bull et al., 2008; Passolunghi et al., 2008) and its visuospatial subcomponents (e.g., Passolunghi and Cornoldi, 2008; Krajewski and Schneider, 2009; Passolunghi and Mammarella, 2010; Alloway and Passolunghi, 2011; Mammarella et al., 2018).

In the original Baddeley and Hitch model (Baddeley and Hitch, 1974; Baddeley, 1998), WM is described as being composed of a unitary component, the central executive (CE), which is in charge of handling and processing information, and by two slave systems, the phonological loop and the visuospatial sketchpad, which is responsible for storing and keeping available verbal and visuospatial information.

The original model structure has been updated (Baddeley, 2000, 2012) and has seen, over time, a series of proposals for change, mainly concentrated on a more detailed specification of the inner structure of the WM components. Logie and Pearson (1997), for instance, proposed to separate the visuospatial notebook into two distinct components: a visual one devoted to the recall of shapes and texture and a spatial one devoted to the recall of spatial location and sequences. Shah and Miyake (1996) questioned the unitary construct of CE, suggesting that a distinction should be made between high-level verbal and visuospatial WM processing.

The Cornoldi and Vecchi (2003) continuity model accepts the suggestions of the literature and depicts WM as a cone structure that develops along two dimensions: a vertical and a horizontal continuum. The horizontal dimension defines the different types of information used in a given task (e.g., verbal, visual, or spatial), hypothesizing that the coding and maintaining of different kinds of information can be processed within semi-independent systems. The vertical continuum represents the level of active control exercised in a given cognitive task, with high control tasks positioned in the upper part of the continuum and simple storage and maintenance tasks in the lower part.

According to the Cornoldi and Vecchi model, it is possible to identify the different components of VSWM: visual WM that allows the temporary storage of visual information (e.g., memory of shapes and colors), spatial WM allowing the recall of positions, (e.g., memory of object positions on a chessboard), or spatial locations presented in sequences, such as for tracking a path (Cornoldi and Vecchi, 2003, see also Mammarella et al., 2008a).

With this model, it is possible to identify both the type of information to be manipulated (e.g., verbal, spatial, visual) and the different levels of cognitive processing involved (active/high control tasks vs. passive/low control tasks) for different WM tasks.

In a recent systematic review, Allen and collaborators (Allen et al., 2019) concluded that a positive effect of visuospatial working memory (VSWM) on mathematics attainments is evident and they suggested that to better understand this relationship, one of the elements to take into consideration is the type of VSWM involved; in the same vein, Mammarella et al. (2018) underlined that only few studies analyzed the relationship between math learning and specific subcomponents of VSWM in typically developing children.

The main body of literature on the involvement of VSWM in early numerical competence and math skills addresses visuospatial short-term memory (STM) tasks, passive/low control tasks in the Cornoldi and Vecchi’s model, such as the forward Corsi block-tapping test or the matrix task that require the recall of positions, but not a true high-level process involving the CE. The results of these works underline the notion that visuospatial STM is implicated during development, both in early childhood numerical performance (McKenzie et al., 2003; Bull et al., 2008) and in initial math ability during the early years of primary school (Gathercole and Pickering, 2000; Simmons et al., 2012).

Some of the works regarding STM and mathematical development have considered the distinction between visual and spatial STM proposed by Logie and Pearson (1997), who explored the relative involvement of visual and spatial STM in math abilities in primary school children (McLean and Hitch, 1999; D’Amico and Guarnera, 2005; Holmes et al., 2008; Mammarella et al., 2010, 2018; Passolunghi and Mammarella, 2010). These studies have confirmed that both visual and spatial STM are implied in math development and suggest that the specific contribution of spatial and visual components could vary by children’s age. The idea of a developmental trend was confirmed by one of the few longitudinal studies following children from preschool age to primary school by Holmes et al. (2008), who evaluated children’s performance in visual and spatial STM tasks and concluded that, while spatial STM is linked to preschool numerical competence level, visual STM is crucial in later years of schooling.

As for the active WM and its involvement in early math performance, the literature is largely associated with the original Baddeley and Hitch model and underlines how the CE, conceived as a unitary structure, is the best predictor of math performance (see Raghubar et al., 2010; Friso-van den Bos et al., 2013 for reviews). Despite the fact that the CE is considered to be the best predictor of mathematics performance in children, CE involvement results come principally from researchers that have used verbal active WM tasks, such as backward digit span, counting recall, and listening span tests, and only a few scholars have studied the relationship between active working memory and math performance, considering the verbal and visuospatial active WM components as being separated. Indeed, most researchers have considered verbal and visuospatial active memory task performances together with a unique score. For instance, in Passolunghi and Lanfranchi’s work (2012), the active WM was evaluated through four different tasks: three verbal WM tasks (a verbal dual task, a phonemic fluency task, and a backward digit span) and a visuospatial dual task, obtaining then a final score calculated from the average of the four single scores. The results showed a direct influence of global active WM on early numeracy abilities in the last year of kindergarten. Similarly, Simmons et al. (2012) investigated the involvement of active WM in early math abilities, measuring WM with a unique method that combined the scores of four complex tasks (two visuospatial dual tasks and two verbal dual tasks), finding that this combined score predicted performance in solving additions in the first year of primary school. These results confirm the involvement of active WM skills in early mathematical learning and performance, but do not allow us to identify the presence of a unique contribution of visuospatial active WM abilities in math abilities development.

The few studies that have kept verbal and visuospatial active WM components distinct suggest that different active WM subcomponents can provide individual contributions to math performance. For example, St Clair-Thompson and Gathercole (2006) examined adolescent’s school performance and detected a strong relationship between visuospatial WM and mathematics achievement while verbal WM was implicated only in English grades. A study by Cornoldi et al. (1995) investigated the academic performance of sixth grade children with low visual–spatial abilities; they found that these children had greater difficulties in mathematics than in other subjects and had greater difficulties in active VSWM tasks compared to passive VSWM tasks. Finally, a longitudinal study of Bull et al. (2008) showed how visuospatial active WM evaluated in first grade students (backward Corsi test) predicted mathematical performance in the third grade of primary school.

Recently, Caviola et al. (2014) examined the role of domain-general and domain-specific precursors of second grade children’s math performance, distinguishing for simple storage WM tasks and complex storage (entailing both storing and processing information) WM tasks.

They found a direct link between complex visuospatial WM tasks and math performance. Szücs (2016) in a recent meta-analysis pointed out that CE functions underlying verbal and visual WM can dissociate and that performance on CE tasks may be modality specific. In the same vein, Mammarella et al. (2018), finding that the difficulties in children with math learning disability (MLD) were related to difficulties in spatial WM passive tasks performance but not in visual ones, concluded that to clarify the role of VSWM it is crucial to cast light on the inner structure of VSWM and on its contribution to math atypical development.

These studies’ results suggest the utility of studying active WM’s contribution to math performance, keeping the visuospatial component apart from the verbal one. As for the question of a possible distinct contribution of visual versus spatial active WM subcomponents to early math development, no researchers have directly investigated the topic to our best knowledge.

In our study, we longitudinally explored the implication on math development of both domain-specific skills during the first 2 years of primary school, taking into account the relationship between early math competence and later math performance, and domain-general variables such as visuospatial active WM abilities.

Specifically, according to the Cornoldi and Vecchi WM continuity model, we investigated the individual contributions of visuospatial active WM subcomponents, keeping distinct the contribution of visual and spatial active WM subcomponents.

Our main purpose was to detect the presence of a distinct contribution of the visual and spatial active WM components toward the children’s math performance from the first to the second grade. We performed a longitudinal design structured in three phases:

1. Time 1 (T1): the beginning of the first class of primary school;
2. Time 2 (T2): the end of first class;
3. Time 3 (T3): the end of second class.

As already mentioned, many scholars have stated that visual and spatial STM processes are linked to math learning, but no literature has been undertaken on the contribution of high-level visual and spatial WM processes on math development, so our predictions are based on the STM literature. If active processing follows the same trend of passive processes (e.g., Holmes et al., 2008), we should expect different contributions from the visual and spatial active WM tasks at different developmental stages, with the spatial abilities most involved in the youngest children’s performance and the visual ones most involved in the subsequent years of schooling. Finally, following the literature showing that early domain-specific abilities, such as numeracy knowledge, are strong predictors for math learning (e.g., Passolunghi et al., 2007), we expect continuity between early numerical competence and math performance during the first 2 years of primary school.

## Materials and Methods

### Participants

The participants consisted of 43 (20 females) typically developing children attending a public school in the town of Cagliari (Italy). The children were tested at the beginning of their first year of primary school (T1: October; mean age = 77.7 months; SD = 3.73; range: 71–84 months), at the end of their first year of primary school (T2: May; mean age = 82.7; SD = 3.73), and at the end of their second primary school year (T3: May; mean age = 92.7; SD = 3.73). All the participants were native Italian speakers. Both the school and the children’s parents agreed to let the students take part in the research study. Informed consent forms were signed by both children’s parents and the school manager, and the local ethics committee granted its approval for the study.

The socioeconomic status of the sample as measured by the Family Affluence Scale (Boyce et al., 2006) was middle class.

### Procedure

The children were individually examined in a single session by an experienced psychologist. The sessions took place in a quiet school room, from 8:30 am to 11:45 am, during school days. Each session lasted about 30 min.

Each participant completed two visuospatial active WM tasks (at T1, T2, and T3), an early numerical competence test (at T1), and a mathematical achievement test (at T2 and T3).

### Materials

#### Visual and Spatial Active Working Memory Tasks

Visual active WM was assessed by the jigsaw puzzle test, and spatial active WM was assessed by the backward Corsi blocks test.

Both the tasks were extracted from an Italian normed visuospatial memory test, the battery for visuospatial memory (BVS)-Corsi test (Mammarella et al., 2008b). Since the BVS-Corsi test is standardized starting from 8 years of age, we performed a pilot study before starting data collection to determine whether the selected tasks were suitable for children as young as the ones in our sample, and we used the tests’ raw scores in our statistical analysis. The same tests were presented at T1 (beginning of the first year of primary school), T2 (end of the first year of primary school), and T3 (end of the second year of primary school).

The jigsaw puzzle test is a task of visual active WM. The task was developed by Vecchi and Richardson (2000) and has been used in the literature to assess active visuospatial WM in old and young people (i.e., Richardson and Vecchi, 2002), and children (i.e., Mammarella et al., 2008a) demonstrating to be a sensitive and reliable tool for investigating active visuospatial abilities.

The test consists of white and black line drawings of common objects (e.g., a lamp, a shoe, or a bicycle) fragmented into parts to form a jigsaw puzzle. The child first sees the drawing of the complete object for 3 s and then is presented with a sheet of paper in which the object is fragmented into randomly arranged numbered parts. The child’s task is to mentally rebuild the original figure by pointing to the different puzzle pieces to indicate how to recreate the original drawing. In the reconstruction phase, the child has 90 s to recreate the original drawing. In this task, the child cannot physically manipulate the puzzle pieces, but can only manipulate them mentally. Before starting the test, a two-piece practice item is shown to the child to verify that the child has understood the instructions. The participants are presented with trials of increasing levels of complexity (from 2 to 10 puzzle pieces) until they are unable to solve at least two out of three items in a level. The final score is the sum of the three most complex items solved.

Spatial WM was assessed through the backward Corsi blocks test. The test consists of a series of nine blocks arranged irregularly on a board. The examiner taps the blocks in a given order, and the child’s task is to watch the examiner and repeat the tapping sequence in backward order.

In the test, the participants are presented with trials of increasing levels of complexity until they are unable to solve at least two trials with the same number of blocks. The backward Corsi span score is determined by the maximum number of blocks correctly recalled in the reverse sequence.

#### Early Numerical Competence Assessment

The children’s early numeracy skills (at T1) were evaluated through the “Battery for the evaluation of numerical intelligence from 4 to 6 years of age” (BIN 4–6) (Molin et al., 2007), a normed test for preschool Italian children. The battery consists of 12 subtests covering four areas: the lexical area (evaluating knowledge of numerical symbols through a numbers recognition task, Arabic digits reading task, and Arabic digits writing task); the semantic area (comparing two Arabic digits to decide which is the largest and comparing two sets of dots to decide which one contains the most dots); the counting area (forward and backward enumerating, ordering Arabic digits, and completing numerical series); and the presyntactic area (matching Arabic numerals with corresponding dots, performing the one-many task, and ordering objects from bigger to smaller and vice versa). The final score of early numerical competence (ENC) is the sum of all the subscales of the battery.

#### Mathematics Achievement

To assess the mathematical performance at T2 and T3, the Italian normed test battery, known as the “Test for the evaluation of calculating and problem-solving abilities” (AC-MT 6–11), was used (Cornoldi et al., 2012). The paper-and-pencil battery was administered collectively to the whole class. The battery consists of the following five subtests:

1. *Operations*: examines the ability to apply calculation procedures. The child must solve additions and subtractions.
2. *Numbers judgment*: evaluates the semantic comprehension of number quantities. For several pairs of Arabic digits, the child must identify the largest number.
3. *Tens and ones task*: evaluates the ability to process the syntactic structure of numbers, building digits from 0 to 99 starting from the number of 10s and ones. For instance, the experimenter says, “Four 10s and two ones correspond to which number?” and, as a response, the child must write the number 42.
4. *Larger to smaller task*: evaluates the semantic representation of numbers. The child must write a random set of displayed numbers in decreasing order.
5. *Small to large task*: this task is similar to the previous one but uses an increasing order.

The final score for mathematics achievement (MA) is the sum of the scores of the five subtests.

### Statistical Analyses

The descriptive statistics of variables and measures were calculated (as reported in Table 1).

**Table 1:** Descriptive statistics of the variables considered in the study for each time (*n* = 43).

	Time 1			Time 2			Time 3		
Variable	Mean	SD	Range	Mean	SD	Range	Mean	SD	Range
Age	77.7	3.73	71–84	82.7	3.73	76–89	92.7	3.73	86–99
Vis-WM	11.19	4.57	2–20	12.12	4.05	2–20	13.53	4.34	4–22
Sp-WM	2.60	0.66	2–5	2.93	0.67	2–5	3.09	0.75	2–5
ENC	100.05	4.27	88–106	–	–	–	–	–	–
MA	–	–	–	20.28	5.25	4–26	23.53	2.64	18–26

*Vis-WM, Visual active WM as measured by the jigsaw puzzle test; Sp-WM, Spatial active WM as measured by the backward Corsi test; ENC, Early numerical competence as measured by the BIN 4–6 test; MA, Mathematics achievement as measured by the AC-MT 6–11 test*.

As a first analysis step, a correlation analysis was carried out, using the Pearson’s correlation index. In the analysis, we examined the relations between the math measures recorded at the three identified times, the BIN 3–6 total score at T1 (ENC_1_), the global AC-MT 6–11 scores at T2 (MA_2_) and T3 (MA_3_), and the relations between visual active WM (Vis-WM) and spatial active WM (Sp-WM) measures and math skills. The alpha level to evaluate the statistical significance of tests was set at 0.05.

Finally, we fitted three regression models to study the influence of the active VSWM variables on early numeracy abilities at T1 (ENC_1_) and on math ability MA at T2 (MA_2_) and T3 (MA_3_).

The relevant predictors were identified by a backward selection procedure based on the Bayesian information criterion (BIC) index (Schwarz, 1978), which selected the best model from a Bayesian perspective.

The analyses were done using R 3.2.2 (R Core Team, 2015).

### Results

Exploratory correlation analyses were performed to examine the relations between math measures and active VSWM measures. The Pearson’s index was used.

From the correlation analysis (Table 2) in which the three numerical abilities evaluated at the three points of time were analyzed together, it appears that the early numeracy abilities evaluated at the beginning of first primary class (ENC_1_) were significantly correlated both with the MA score at the end of the first class (MA_2_) (*r* = 0.51; *p* < 0.001) and with the score of the MA at the end of the second class (MA_3_) (*r* = 0.37; *p* = 0.014). Moreover, MA_2_ was significantly correlated with MA_3_ (*r* = 0.33; *p* = 0.028).

**Table 2:** Pearson’s r correlation between active VSWM measures and early numerical competence tasks at T1 and numerical intelligence at T2 and T3 (*n* = 43).

Measure	1	2	3	4	5	6	7	8	9
1. ENC_1_	–								
2. MA_2_	0.51***	–							
3. MA_3_	0.37*	0.33*	–						
4. Vis-WM(T1)	0.36*	0.40**	0.05	–					
5. Sp-WM(T1)	0.46**	0.35*	0.18	0.28	–				
6. Vis-WM(T2)	0.52***	0.31*	0.15	0.40**	0.47**	–			
7. Sp-WM(T2)	0.35*	0.23	0.30*	0.28	0.26	0.24	–		
8. Vis-WM(T3)	0.29	0.28	0.32*	0.58***	0.32*	0.45**	0.27	–	
9. Sp-WM(T3)	0.26	0.21	0.38*	0.10	0.22	0.19	0.39**	0.01	–

*ENC_1_, Early numerical competence at T1; MA_2_, Mathematics achievement at T2; MA_3_, Mathematics achievement at T3; Vis-WM(T1), Visual active WM at T1; Sp-WM(T1), Spatial active WM at T1; Vis-WM(T2), Visual active WM at T2; Sp-WM(T2), Spatial active WM at T2; Vis-WM(T3), Visual active WM at T3; Sp-WM(T3), Spatial active WM at T3*.

* *p < 0.05*;

** *p < 0.01*;

*** *p < 0.001*.

Moreover, the analysis showed significant correlations between ENC_1_ and Vis-WM (T1) [*r* (43) = 0.36, *p* < 0.05] and Sp-WM (T1) [*r* (43) = 0.46, *p* < 0.01]. Further, significant correlations were also found between MA_2_ and Vis-WM (T1) [*r* (43) = 0.40, *p* < 0.01], Sp-WM (T1) [*r* (43) = 0.35, *p* < 0.05], and Vis-WM (T2) [*r* (43) = 0.31, *p* < 0.05]. No significant correlation was observed between Sp-WM (T2) and MA_2_ [*r* (43) = 0.23, *p* > 0.05]. Finally, significant correlations were also found between MA_3_ and Sp-WM (T2) [*r* (43) = 0.30, *p* < 0.05], Vis-WM (T3) [*r* (43) = 0.32, *p* < 0.05], and Sp-WM (T3) [*r* (43) = 0.38, *p* < 0.05].

As a second analysis step, we fitted three regression models to study the influence of active VSWM variables both on early numeracy abilities at T1 (ENC_1_) and on math ability at T2 (MA_2_) and T3 (MA_3_). For each time, we proceeded as follows. A full model, which considers all the variables as predictors, has been analyzed through a backward selection procedure based on the Bayesian Information Criterion (BIC) index (Schwarz, 1978) which selects the best model in a Bayesian perspective. Using the full model as reference, we deleted one by one the predictors. At each step of deletion, we compared the BIC of each comparison model with the full model’s BIC, looking for an improvement and calculating the difference between the new model and the reference model (DBIC). When relevant predictors were identified, we fitted a new reference model and tried one more time to delete predictors, looking for further improvements. The procedure stopped when the best fit was obtained. Moreover, we calculated the DBIC between the best fit model and the null model (a model without any predictors) to evaluate how much the predictors affected the dependent variable. See Table 3 for details.

**Table 3:** Models’ selection details: null model, full model, best fit model for each time and related BIC indexes and DBIC values.

Time	Model	BIC	DBIC
T1	Null model (ENC_1_ ~ 1)	128.54	(A) −6.62
	Full model (ENC_1_ ~ VisWM_1_ + SpWM_1_)	122.37	(B) −0.45
	Best fit model (ENC_1_ ~ SpWM_1_)	121.92	
T2	Null model (MA_2_ ~ 1)	128.54	(A) −8.96
	Full model (MA_2_ ~ ENC_1_ + VisWM_1_ + SpWM_1_ + VisWM_2_ + SpWM_2_)	130.64	(B) −11.07
	Best fit model (MA_2_ ~ ENC_1_)	119.57	
T3	Null model (MA_3_ ~ 1)	128.54	(A) −4.94
	Full model (MA_3_ ~ ENC_1_ + MA_2_ + VisWM_1_ + SpWM_1_ + VisWM_2_ + SpWM_2_ + VisWM_3_ + SpWM_3_)	134.95	(B) −11.35
	Best fit model (MA_3_ ~ VisWM_3_ + SpWM_3_)	123.60	

*DBIC (A), Difference between best fit model’s BIC and null model’s BIC; DBIC (B), Difference between best fit model’s BIC and full model’s BIC*.

The results at T1 (full model, dependent variable: ENC_1_; predictors: Vis-WM at T1 and Sp-WM at T1) showed that the best model is the one in which ENC_1_ was predicted only by the spatial active WM task, which explained 21% of the variance in early numeracy ability. The results at T2 (full model, dependent variable: MA_2_; predictors: T1 and T2 Vis-WM and T1 and T2 Sp-WM and ENC_1_) showed that the best model is the one in which MA_2_ performance was predicted only by the ENC_1_ measured at T1 which explained 25% of the variance in mathematics achievement. The results at T3 (full model, dependent variable: MA_3_; predictors: T1, T2, and T3 Vis-WM; T1, T2, and T3 Sp-WM; ENC_1_; and MA_2_) showed that the best model is the one in which MA_3_ performance was predicted by both the visual and spatial active WM measured at T3, which together explained 25% of the variance in mathematics achievement at the end of the second year of primary school. See Table 4for details.

**Table 4:** Best fit model for each time.

Dependent variable	Predictors	*β*	Std. error	*t*	*p*	Total *R*^2^
Time 1						
ENC_1_	Sp-WM_1_	0.46	0.14	3.34	0.001	0.21
Time 2						
MA_2_	ENC_1_	0.50	0.13	3.75	0.0004	0.25
Time 3						
MA_3_	Vis-WM_3_	0.32	0.14	2.80	0.022	0.25
	Sp-WM_3_	0.38	0.14	2.36	0.007	

*ENC_1_, Early numerical competence at T1; MA_2_, Mathematics achievement at T2; MA_3_, Mathematics achievement at T3; Sp-WM(T1), Spatial active WM at T1; Vis-WM(T3), Visual active WM at T3; Sp-WM(T3), Spatial active WM at T3*.

## General Discussion

In this paper, we used a longitudinal design to study the involvement of active VSWM on early numeracy abilities during the first 2 years of primary school. In our work, we have distinguished between spatial and visual active WM subcomponents and took into account the relationship between active WM subcomponents and domain-specific math skills.

The children at the beginning (T1) and end (T2) of first grade and at the end of second grade (T3) were given two tasks measuring visual active WM (jigsaw puzzle task) and spatial active WM (backward Corsi task). Moreover, early numeracy abilities were measured at the beginning of the first year of primary school with an early numerical ability normed test (BIN 4–6) (Molin et al., 2007), while mathematical skills were measured at the end of the first and second years of primary school with a normed math test (AC-MT 6–11) (Cornoldi et al., 2012).

From the correlation analysis, performed to explore the relationship between math measures and active VSWM measures, we observed that early numeracy competences (BIN test score at T1) significantly correlated over time with later math skills (total AC-MT 6–11 test score) at both T2 and T3. The children who had best learned how to handle numerical magnitude and numerical symbols before starting school showed—at the end of both first and second grades—a better comprehension of the semantic and syntactic structure of numbers and were more skilled in written calculations. This was an expected result: many studies have shown that early deficiencies affect the entire schooling path, especially for mathematics (Geary, 1993, 2013; Geary et al., 1999, 2000), and that the level of early numeracy abilities is one of the best predictors of school success in mathematics (Gersten et al., 2005; Jordan et al., 2006; Passolunghi et al., 2007, 2008; Lyons et al., 2014).

Concerning the relationship between VSWM and math skills at the three time points, the correlation analysis showed a strong relationship between both visual and spatial WM skills and early knowledge of magnitude and numbers at the beginning of first grade, while at the end of first grade, we found that only visual active WM was related to the math test score and that it was only at the end of second grade that both visual and spatial active WM came back into play.

As for the developmental trend along the three times, the correlation analysis showed that the VSWM abilities of the children at the beginning of first grade were correlated with their math performance measured at the end of the first grade, but not with their math abilities measured after 1 year. These results suggested that the VSWM skills we tested at all three time points were related to the magnitude/numerical knowledge and calculation ability of the children, but with a relative different weight depending on the considered period.

To better explore these results, three regression analysis were computed; each analysis had performance in mathematics at one of the three time points as its dependent variable and all the domain-specific and domain-general variables measured up to that time as predictors.

The results confirmed that it is possible to find a different specific involvement of domain-specific and domain-general variables at different time steps. At the beginning of the first grade, spatial active WM is the best predictor of early numeracy abilities (with 21% of the variance explained). Therefore, at the very beginning of primary school, when formal math teaching has not yet occurred, the construction of basic knowledge about magnitudes and numbers and the first knowledge about the lexical and semantic aspects of numbers are strongly influenced by spatial active WM. Moving to the end of first grade, the second regression analysis showed that the only influence on math performance at this early stage of formal math learning comes from early numerical knowledge as measured at T1, while the domain-general variables’ influence disappears. In this stage, the influence of the already owned knowledge about quantities and numerals was so strong that almost all the variances of the model were explained by the BIN test score measured at T1 (25%). The two math tests we used in the study are very correlated and BIN test is considered a good predictor of later math skills measured with ACMT (see BIN manual for details, Molin et al., 2007). This relationship is well confirmed by our findings.

The results of the third regression analysis, in which the dependent variable was the children’s math performance at the end of the second class, again showed a changed situation in which the best predictors of math performance were both visual and spatial WM (respectively with *β* = 0.32, *p* = 0.022; and *β* = 0.38, *p* = 0.007) as measured at T3.

In summary, our regression models outlined a situation in which, at the very beginning of familiarization with numeracy (T1), the ability to store and process spatial configurations was crucial to help children familiarize themselves with quantity and number concepts. At the end of the first grade (T2), instead, the child’s performance was so strongly founded on previous domain-specific knowledge that the performance on the early numerical competence test was the only significant predictor. Finally, both visual and spatial WM abilities were strongly implicated in later math achievement at the end of second grade (T3), when the children (in the Italian school context) faced more complex math tasks, such as additions and subtractions with two digits, and began to manage 10s and ones and their relative placement.

From a developmental point, the fact that we found different specific contributions of the domain-general and domain-specific variables engaged with the math performance at different stages of the learning path is interesting because it shows that in different moments, even very close in time, children can rely on different resources for seemingly similar tasks.

Our exploratory study aimed mainly to cast light on the involvement of visual and spatial active WM in the first phases of math learning, at a developmental stage for which no data on this specific topic are available: the first 2 years of primary school. Even considering the limitation of a relatively scarce sample size and the fact that for each VSWM subcomponent, only one task has been used, our results showed that active VSWM starts to influence math performance from the very first phases of math learning and that the different individual contributions of visual and spatial components can be identified.

As previously mentioned, to our knowledge, no studies have been performed with such young children indicating the relationship between math skills development and visual and spatial active WM subcomponents, so our expectations for this study were mainly based on passive WM literature. Our results follow the same trend observed by researchers that have studied the influence of the spatial and visual passive memory components on early numerical cognition: both our work and passive WM studies have shown a greater involvement of the spatial component than the visual one on pre- and early math abilities. For example, McKenzie et al. (2003) found that 6-year-olds mainly use a spatial rather than a visual-type strategy to solve arithmetic operations. Holmes et al. (2008) found that primary school children initially rely on spatial strategy to solve math problems and suggested that spatial (passive) WM functions as a workspace to support the transition from early concrete informal knowledge to the nascent formal mathematical knowledge. Our results, therefore, confirm the passive memory literature showing that the link between numbers and space is particularly important for the development of numerical cognition and showed that spatial active WM contributes to the formation of the first, mostly spatial, numerical representations at a very early stage of math learning.

The fact that we found a different involvement of the spatial and visual active WM components at different ages confirmed the importance of distinguishing between visual and spatial WM components at the processing level, following the suggestion from the passive WM literature (e.g., McKenzie et al., 2003), and evoked interesting questions even at a more general theoretical level. Following the conical continuity model of WM (Cornoldi and Vecchi, 2003), the differentiation between the various domain-specific systems, including visual and spatial information, are more clearly marked in the simple-storage area at the base of the cone, and this feature could explain why the passive literature was able to find differences between these components. The fact that our findings, even using high control tasks, discern different implications of visual and spatial abilities at different time steps suggests that even at a higher level of control (rising into the cone structure), these two abilities are sufficiently differentiated, at least in regard to their involvement in math development.

Given that in our analysis we have used math competence global scores obtained by the sum of different subtests, our study is not able to determine the degree of relationship of the individual math ability components to the visuospatial processes. As a suggestion for future research, it would be interesting to examine the detail of the individual contribution of different math abilities to the VSWM subcomponents in early math development stages, in line with the recent literature investigating older children (e.g., Caviola et al., 2014; Mammarella et al., 2018; see Allen et al., 2019 for review).

The literature regarding active VSWM involvement in young children’s cognitive performance and specifically in early math development is too scarce at the moment for more precise predictions, but the topic is worthy of further investigation.

## Conclusions

Our exploratory longitudinal study showed that both domain-specific knowledge and domain-general variables are implied in the initial math learning process, but with different weights along time. The prerequisites confirmed themselves as the foundation of initial math learning at the end of first grade, while domain-general variables (e.g., visual and spatial WM abilities) play an important role in math learning processes before the beginning of first grade and after the end of second grade, with different weights at different moments.

This result suggests the importance of distinguishing between visual and spatial WM components at the theoretical and processing levels (and not only at the simple storage level) and implies that the involvement of general domain abilities in early math performance can be better understood within the framework of active WM models in which the spatial and the visual components are kept separate, such as in the WM continuity model (Cornoldi and Vecchi, 2003).

The relationships between active VSWM variables and initial math learning as highlighted in our study are important for a better understanding of the components that are in play in the first phases of math abilities development and for better identifying strengths and weaknesses in children with early math difficulties. These relationships are worth investigating in future longitudinal research involving a larger number of subjects and tasks and extended for a longer time span.

## Data Availability Statement

The raw data supporting the conclusions of this manuscript will be made available by the authors, without undue reservation, to any qualified researcher.

## Ethics Statement

The studies involving human participants were reviewed and approved by Comitato etico del Dipartimento di Pedagogia, Psicologia, Filosofia – Università degli Studi di Cagliari. Written informed consent to participate in this study was provided by the participants’ legal guardian/next of kin.

## Author Contributions

RF and CM were involved in the planning of the study. DM and CM did the data analysis. CM wrote the “Introduction” section. DM and CM wrote the “Materials and Methods” and “Results” sections. RF wrote the “General Discussion” and “Conclusions” sections of the manuscript, and revised the “Introduction,” “Materials and Methods,” and “Results” sections.

### Conflict of Interest

The authors declare that the research was conducted in the absence of any commercial or financial relationships that could be construed as a potential conflict of interest.
